# Competition-driven evolution of organismal complexity

**DOI:** 10.1371/journal.pcbi.1007388

**Published:** 2019-10-03

**Authors:** Iaroslav Ispolatov, Evgeniia Alekseeva, Michael Doebeli

**Affiliations:** 1 Departamento de Fisica, Universidad de Santiago de Chile, Santiago, Chile; 2 Center of Life Sciences, Skolkovo Institute of Science and Technology, Moscow, Russia; 3 Department of Zoology and Department of Mathematics, University of British Columbia, Vancouver B.C. Canada; University of Illinois at Urbana-Champaign, UNITED STATES

## Abstract

Non-uniform rates of morphological evolution and evolutionary increases in organismal complexity, captured in metaphors like “adaptive zones”, “punctuated equilibrium” and “blunderbuss patterns”, require more elaborate explanations than a simple gradual accumulation of mutations. Here we argue that non-uniform evolutionary increases in phenotypic complexity can be caused by a threshold-like response to growing ecological pressures resulting from evolutionary diversification at a given level of complexity. Acquisition of a new phenotypic feature allows an evolving species to escape this pressure but can typically be expected to carry significant physiological costs. Therefore, the ecological pressure should exceed a certain level to make such an acquisition evolutionarily successful. We present a detailed quantitative description of this process using a microevolutionary competition model as an example. The model exhibits sequential increases in phenotypic complexity driven by diversification at existing levels of complexity and a resulting increase in competitive pressure, which can push an evolving species over the barrier of physiological costs of new phenotypic features.

## Author summary

We provide a theoretical proof-of-principle explanation for the empirically well-documented macroevolutionary pattern of punctuated changes in organismal complexity. According to such patterns, many phenotypic properties related to organismal complexity, such as body size, the development of new sensory or locomotive capabilities, etc., show long periods of relatively slow changes interrupted by intermittent bursts of rapid evolutionary changes. Building on earlier work, we argue that punctuated expansions in organismal complexity can be caused by a threshold-like response to growing ecological pressures in diversifying communities at an existing level of complexity. Acquisition of novel phenotypic features then allows a species to escape this pressure once the existing diversity reaches a certain threshold. Continuing our previous line of research on microevolutionary models for long-term evolution in high-dimensional phenotype spaces, we present a detailed quantitative description of this process based on a classic competition model. The resulting macro-evolutionary dynamics exhibit sequential increases in phenotypic complexity driven by the build-up of competitive pressures at an existing level of complexity, which allows the evolution of novel phenotypes despite substantial physiological costs.

## Introduction

The notion of “complexity” in biological organisms is often rather imprecise and intuitive [1] and generally implies that one organism is more complex than another if it has more diverse and better regulated metabolic functions, more advanced sensory capabilities, additional means of locomotion in diverse physical environments, etc. In this context, a reasonable proxy for the complexity of a multicellular organism would e.g. be the number of different tissues in its body plan, or the number of cell types, or the number of transcriptional regulatory programmes [1]. An increase in complexity would normally entail energy costs for reproduction and development, but as a result of increased complexity, organisms can achieve significant ecological and, on larger time scales, evolutionary advantages. In this work, we quantify complexity as a number of phenotypic traits or dimensions, so an increase in complexity corresponds to expansion into previously unoccupied phenotypic dimensions. Double fertilization in flowering plants, breathing of atmospheric oxygen in amphibians, separation of blood circulation in reptiles, and development of speech in humans are just a few examples of such evolutionary increases in organismal complexity [2] (sometimes called aromorphosis).

Substantial knowledge has been accumulated about the mechanisms of diversification within many different groups, representing different levels of complexity. However, still little seems to be known about the evolution of significant innovations and the associated evolutionary changes in complexity [3, 4]. What seems clear is that the evolution of complexity takes time: about 3 billion years passed between the emergence of life and the appearance of the first multicellular organisms, the first land-based species only appeared about 450 millions years ago, and the first known flying creatures (insects), appeared a mere 350 million years ago.

It has been argued that significant increases in organismal complexity that open a new ecological niche should often be followed by rapid diversification and subsequent saturation of diversity in that niche (e.g [5, 6, 7]). The rates of evolutionary change and of speciation is expected to be high when a niche is newly formed and almost empty, and to decrease as the niche gets filled through diversification [6, 7]). Such patterns are sometimes referred to as “punctuated equilibrium” or “blunderbuss pattern” [8, 5], indicating that a uniform accumulation of mutations could not account for intermittent evolutionary bursts. However, what sets the pace of such repeating bursts remains unclear. A number of times in Earth’s history, the formation of new niches was caused by geological or cosmic catastrophes, yet it also seems highly likely that such patterns can be caused by intrinsic dynamics of the evolving biosphere itself.

Evolutionary increases in complexity are expected to be slow. They depend on two processes. On the one hand, evolution of the phenotype is impossible without mutagenesis. Mutations occur with certain probabilities at certain locations in the genome. Single nucleotide substitutions are the most common type of mutational event, and most of the genetic differences within a population are due to such polymorphisms. However, single nucleotide substitutions tend to have small phenotypic effects, and the action of selection at such a scale may be limited [9]. In contrast, other mutational events, such as gene duplications or chromosomal rearrangements, are probably rarer, but potentially have much larger phenotypic effects and may lead to the evolution of novel phenotypes [10, 11, 12]. Indeed, genomic analysis of closely related species shows that such mutations seem to be important in the history of aromorphosis [13]. The transition to a new level of phenotypic complexity requires accumulation of a set of particular mutations that usually have to come in a certain sequence to be useful and get fixed. Those sequences of specific mutations can have very low probabilities and thus require long waiting times to occur.

On the other hand, it seems reasonable to think that an increase in complexity would entail physiological costs for reproduction and development, but that as a result of increased complexity, organisms can gain significant ecological advantages. In particular, selection is expected to be very important for the evolution of complexity. Indeed, selection may depend on prevalent ecological pressures, and when these pressures increases beyond a certain threshold, e.g. due to the evolution of a high level diversity occupying the currently available niches, the cost of additional complexity may turn out to be less than the advantage of exploiting a novel ecological niche, in which there may be less competition and/or predation.

Here we focus on the role of ecological processes for increase in complexity and provide conceptual and numerical illustrations that competition can in principle drive such evolutionary processes. Based on logistic competition models, we quantitatively explore how the intensity of ecological interactions can drive increased organismal complexity despite physiological costs. In our models, increases in organismal complexity are described as new dimensions in phenotype space that are acquired during evolution, while the level of diversity is reflected by the number of distinct species. The physiological cost of adding a new phenotypic dimension is implemented as a reduction in birth rates, while the competitive advantage gained as a result of such addition is modeled as an increase in the environmental carrying capacity and as an initial relief from competition. We show that when the physiological costs of adding a new phenotypic capability are comparable to the benefits that a carrier of the corresponding fully developed capability can gain, then the initial increase in complexity, i.e., the initial gradual acquisition of the new phenotypic dimension, can indeed be driven by ecological interactions.

## Methods

### The model

To model the evolution of complexity due to ecological interactions, we study a general class of models for frequency-dependent competition [14, 15, 16, 7], in which ecological interactions are defined by continuous *d*-dimensional phenotypes, where *d* ≥ 1. For example, one can imagine that dimensions in phenotypes **x** of individuals are given by the efficiencies of several metabolic pathways, or various morphological characteristics. An acquisition of a new phenotypic capability is viewed as an expansion into a new phenotypic dimension that represents the new phenotype.

Competitive ecological interactions that define the logistic model are determined by a competition kernel *α*(**x**, **y**) and a carrying capacity *K*(**x**), where **x**, **y** are the phenotypes of competing individuals. The competition kernel *α*(**x**, **y**) measures the competitive impact that an individual of phenotype **x** has on an individual of phenotype **y**, and in the sequel we always assume that *α*(**x**, **x**) = 1 for all **x**. To take into account the physiological cost of maintenance of new phenotypes, we extend the model considered in [14, 15, 7] by adding a phenotype-dependent birth rate *β*(**x**). Then the logistic ecological dynamics for individual with phenotype **y** in the environment with individuals with phenotypes **x**_**p**_ is completely determined by the birth rate *β*(**y**) and the death rate

$$\begin{array}{c} \frac{\sum_{p}\alpha \left(x_{p},y\right)}{K(y)}. \end{array}$$ (1)

To make our arguments clearer, and to simplify the analysis, we apply the standard adaptive dynamics approach [17, 18], which describes the evolution of a population cluster as the motion of a group of individuals with identical phenotypes in phenotypic space. The invasion fitness, i.e., the per capita growth rate of a rare mutant with phenotype **y** in the resident monomorphic population with phenotype **x** and ecological equilibrium population density *K*(**x**), is given by

$$\begin{array}{c} f\left(x,y\right)=\beta \left(y\right)-\frac{\alpha (x,y)K(x)}{K(y)}. \end{array}$$ (2)

With the resident assemblage consisting of several phenotypic species *r* = 1, …, i.e. subpopulations that are monomorphic for a given phenotype **x**_**r**_ (also referred to as population clusters, or simply clusters), the adaptive dynamics for a species with phenotype **x** is determined by its selection gradient **s**(**x**) with components

$$\begin{array}{c} s_{i}\left(x\right)\equiv \frac{\partial f(x,z)}{\partial z_{i}}{|}_{z=x}=\frac{\partial \beta (x)}{\partial x_{i}}-\sum_{r}\frac{N_{r}}{K(x)}\frac{\partial \alpha (y_{r},z)}{\partial z_{i}}{|}_{z=x}+\sum_{r}\frac{\partial K(x)}{\partial x_{i}}\frac{\alpha \left(y_{r},x\right)N_{r}}{K^{2}\left(x\right)}, \end{array}$$ (3)

(see [19, 14, 15, 7] for more details). Here *N*_*r*_ is the equilibrium population size of the cluster with phenotype **y**_**r**_, which is given by the stationary solution of the system of logistic population dynamics equations,

$$\begin{array}{c} \frac{dN_{r}}{dt}=N_{r}[\beta \left(y_{r}\right)-\frac{\sum_{r^{\prime }}\alpha \left(y_{r^{\prime }},y_{r}\right)N_{r^{\prime }}}{K(y_{r})}]. \end{array}$$ (4)

The selection gradients define a system of differential equations in phenotype space $R^{d}$, each describing the evolution of phenotype **x**_*r*_ of population cluster *r* with population density *N*_*r*_

$$\begin{array}{c} \frac{dx_{r}}{dt}=N_{r}s_{r}\left(x_{r}\right). \end{array}$$ (5)

For simplicity and generality, here we assumed that the mutational variance-covariance matrix for each cluster, which reflects peculiarities of genotype-phenotype mapping, is diagonal with elements equal to the population size of the corresponding cluster. This corresponds to the assumption that mutations occur independently in all phenotypic directions, with equal average size and at equal per capita rates. More details on the derivation of the adaptive dynamics (5) can be found in a large body of original literature (e.g. [17, 18, 20, 21, 19]).

The standard adaptive dynamics is extended as in [7] to include diversification, which manifests itself as the splitting of clusters. Each *τ*_*c*_ ∼ 1 time units a contingent new cluster is created by randomly picking an existing cluster, splitting it in half, and separating the two new clusters in a random direction in phenotype space by a distance Δ*x* ∼ 10^−3^. The newly created cluster evolves as all other existing clusters, however, just before the next splitting event, the distances between clusters are assessed and those which are closer to each other than a threshold Δ*x* are merged. This ensures a randomly assigned capability for all populations to diversify: if a chosen population cluster is under selection to undergo diversification, the split halves will diverge sufficiently so that they will not be merged back at the next check. Alternatively, when a cluster splits that is not under diversifying selection, the two halves will not diverge and instead will be merged again at the next check. We observed that clusters, once separated by the distance ∼ Δ*x*, almost never come close to each other or other clusters again, so merging of either previously unrelated clusters or of split clusters after several intervals *τ*_*c*_ is highly unlikely.

The key parts in our model are the definitions of the birth rate *β*(**x**), the competition kernel *α*(**x**, **y**), and the carrying capacity *K*(**x**), which reflect the costs and advantages of acquiring new phenotypes. An acquisition of an additional phenotypic feature should result in certain benefits: we express those benefits as an increase in the carrying capacity, resulting in a reduced death rate. However, it should also be accompanied by a cost, reflecting higher physiological costs of maintaining a more complex organism. Here we incorporate these costs in the birth rate. Taking these factors into account, our model works as follows:

- A cluster is considered lacking the phenotypic dimension *i* when the corresponding phenotypic coordinate *x*_*i*_ is close to zero. For example, one can think of a certain phenotypic dimension as of an ability to metabolize a particular substance, so that the corresponding phenotypic coordinate is the rate of this metabolic process. The inability to metabolize a substance means that the rate of corresponding process is zero.
- A single-humped shape of the environmental carrying capacity corresponds to the simplest and probably often realistic scenario of a particular combination of those abilities being optimal in the absence of competition. Thus we chose the definition of carrying capacity similarly to [14, 15, 16, 7],
  $$\begin{array}{c} K\left(x\right)=\text{exp}[-\frac{\sum_{i=1}^{d}{\left(x_{i}-C\right)}^{4}}{4}], \end{array}$$ (6)
  but with the maximum at **x** = **C** ∼ 1. We consider that a cluster acquires a particular phenotypic dimension *i* when *x*_*i*_ moves away from zero and gets sufficiently close *C*. Doing so, the organism makes full use of its new phenotypic capability by maximizing the carrying capacity in that phenotypic dimension.
- The initially existing simplest life is represented by a single cluster that has only one phenotypic dimension, and the birth rate is the same for all phenotypes along this dimension.
- Each transition to a higher phenotypic dimension is associated with a cost implemented as a reduction in the birth rate. We define the birth rate *β*(**x**) as
  $$\begin{array}{c} \beta \left(x\right)=\prod_{i=2}^{d}\{\text{exp}[-\frac{{\left(x_{i}\right)}^{2}}{2\sigma_{\beta }^{2}}]\left(1-b\right)+b\}. \end{array}$$ (7)
  The first phenotypic dimension does not carry any birth rate penalty, thus the product in (7) starts with *i* = 2. When a new dimension is acquired, that is, the corresponding coordinate changes from almost zero to a value much larger than the birthrate penalty width *σ*_*β*_, the birthrate is multiplied by a factor *b* < 1.
- For simplicity, we use a symmetric Gaussian competition kernel
  $$\begin{array}{c} \alpha \left(y,x\right)=\text{exp}[-\frac{\sum_{i=1}^{d}{\left(x_{i}-y_{i}\right)}^{2}}{2\sigma_{\alpha }^{2}}], \end{array}$$ (8)
  in which the competitive effect of **y** on **x** is equal to that of **x** on **y**. This form of competition kernel also promotes expansion into new phenotypes as the multiplicative nature of (8) ensures that the competition gets weaker if any of the distances |*x*_*i*_ − *y*_*i*_| increases. For instance, this happens when a cluster acquires the dimension *i*, so that *x*_*i*_ ∼ *C*, while the rest of the system does not, so that *y*_*i*_ ≈ 0.

### Adaptive dynamics of acquisition of a new phenotype

Let us consider a generic scenario of a competition-driven expansion into a higher phenotypic dimension. For simplicity of visualization, we consider the evolutionary transition from 1-dimensional to 2-dimensional phenotype space. However, the same arguments apply to any increase in phenotypic dimension. Two components of the selection gradient (3) for a single population cluster initially living in the first dimension are

$$\begin{array}{c} s_{1}\left(x_{1},x_{2}\right)=-{\left(x_{1}-C\right)}^{3}\{\text{exp}[-\frac{{\left(x_{2}\right)}^{2}}{2\sigma_{\beta }^{2}}]\left(1-b\right)+b\}\\ s_{2}\left(x_{1},x_{2}\right)=-\frac{x_{2}}{\sigma_{\beta }^{2}}\text{exp}[-\frac{x_{2}^{2}}{2\sigma_{\beta }^{2}}]\left(1-b\right)-{\left(x_{2}-C\right)}^{3}\{\text{exp}[-\frac{{\left(x_{2}\right)}^{2}}{2\sigma_{\beta }^{2}}]\left(1-b\right)+b\}. \end{array}$$ (9)

Selecting a sufficiently small width *σ*_*β*_ in the birthrate penalty term makes the contribution of this term dominant and restricts the evolutionary dynamics of the single cluster to a narrow strip along the *x*_1_ axis, *x*_2_ ≪ *C*. In this case, evolution in the first phenotypic dimension follows the standard adaptive dynamics scenario, i.e. the single cluster moves to the center of the carrying capacity $x_{1}^{*}=C$ and then, for sufficiently small *σ*_*α*_, evolutionary branching occurs [22, 23] with subsequent diversification into two different phenotypic clusters (with each one still having a 1-dimensional phenotype). After splitting, each cluster experiences an additional contribution $\tilde{s}$ to its selection gradient that is produced by the gradient of the competition kernel and is given by the second term in the right-hand side of (3). If the two new clusters have phenotypes **x** and **y** and the distance between them, |**x** − **y**|, is sufficiently small, the components of this contribution are

$$\begin{array}{c} {\tilde{s}}_{i}\left(x\right)=\frac{x_{i}-y_{i}}{2\sigma_{\alpha }^{2}}\text{exp}[-\frac{\sum_{k=1}^{2}{\left(x_{k}-y_{k}\right)}^{2}}{2\sigma_{\alpha }}],\\ {\tilde{s}}_{i}\left(y\right)=-{\tilde{s}}_{i}\left(x\right),\;i=1,2. \end{array}$$ (10)

The factor 2 in the denominator appears because the population of each of the recently separated clusters is approximately half of the carrying capacity. Assume that for a typical separation |**x** − **y**|, the addition of (10) to the second-dimensional component of the selection gradient tilts the balance and makes the *s*_2_ positive for one of two new clusters. This cluster will start moving in the positive *x*_2_ direction. At the same time, the ${\tilde{s}}_{1}$ components of (10) is pushing the two clusters apart in the *x*_1_ dimension (again under the assumption that *σ*_*α*_ is small enough to produce such diversification). That, in turn, will reduce the exponential factor in ${\tilde{s}}_{2}$ (10), which depends multiplicatively on both |*x*_1_ − *y*_1_| and |*x*_2_ − *y*_2_|, and at some point will turn the component *s*_2_ negative, resulting in a failed attempt to increase phenotypic complexity. Such failure thus occurs when diversity in the existing phenotypes is not high enough and the newly split clusters have ample space to diverge along the *x*_1_ coordinate.

Consider now a complimentary scenario when, as a result of repeated diversification, [7], the *x*_1_ dimension has become saturated with phenotypic clusters, so that further diversification in the *x*_1_-direction is impossible. This implies that when a new cluster is formed, there will be no net repulsive *s*_1_ component in the selection gradient acting on it. Any such component from a nearest neighbour will be cancelled by competitive repulsion from other clusters. However, evolution of a newly formed cluster with *x*_2_ > 0 in the positive *x*_2_ direction will not be impeded. Rather, there is competitive pressure to evolve away from *x*_2_ ≈ 0 due to the sum of competitive contributions to *s*_2_ generated from all the clusters present in the *x*_1_-direction, since their *y*_2_ components are close to zero and thus less than *x*_2_ (see (10)). The resulting positive contribution to the selection gradient can exceed the negative part of the selection gradient in the *x*_2_-direction that is caused by the cost in the birth rate. Once the competition from the clusters present in the *x*_1_-direction has pushed the *x*_2_-component of the new cluster sufficiently far away from 0, so that *x*_2_ ≫ *σ*_*β*_, the negative part of the selection gradient coming from the cost in the birth rate disappears, and *x*_2_ continues to increase further and converges to *C*, driven by both an increase in the carrying capacity and by competition from the other clusters.

The essential mechanism underlying the above scenarios is that once diversity has saturated in a given dimension, the resulting selection pressure due to competition can be enough to drive evolution into new phenotypic dimensions despite the necessity of overcoming a negative fitness gradient that is due to costs of the initial development of the new phenotypes. These costs are high enough such that without saturation, competition simply results in further diversification along the already existing phenotypic dimension. In principle, this should result in a two-tiered evolutionary process: first, there is diversification along existing phenotypic dimensions; once this diversification has saturated, i.e., once the niches along existing phenotypes are sufficiently full, competitive pressures from the saturated community facilitate the evolution of new phenotypic dimensions that are “orthogonal” to existing ones. Acquisition of this new phenotypic dimension should then be followed by another round of repeated diversification in the newly established phenotype space, eventually leading to saturation, which then in turn can again generate further increases in phenotypic complexity. The rather complex interplay between the carrying capacity, the birth rate and the competition kernel in our models do not allow us to make these argument more precise mathematically, but the numerical examples shown in the next two sections indicate that such scenarios can indeed be realized for a range of parameters *σ*_*β*_, *b*, *σ*_*α*_, and *C*.

## Results

### Transition from one-dimensional to two-dimensional systems

**Fig 1:**
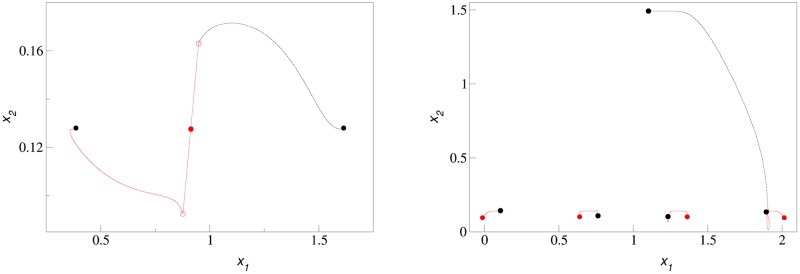
Adaptive dynamics trajectories of diversifying clusters showing failed (left) and successful (right) expansion into the second dimension. Left panel: Low diversity scenario of splitting of a single one-dimensional cluster (shown by a solid red circle) into two halves (shown by two empty red circles), which move apart but stay in the first dimension (red and black lines terminating with black circles, which show the equilibrium positions of new clusters.) The splitting distance is artificially enlarged to show that even that does not lead to expansion into new dimension. Right panel: Saturated diversification scenario when the rightmost of four 1-dimensional clusters (whose adaptive dynamics trajectories start from solid red circles and are shown with red lines) is split into halves and one half moves up into the second dimension (its trajectory is shown as black dashed line), while the other half stays in the first dimension, evolving along a loop-like trajectory shown as a red solid line. Black circles show the final equilibrium positions of resulting five clusters. The parameters are *σ*_*β*_ = 0.15, *b* = 0.84, *C* = 1, and *σ*_*α*_ = 0.5.

Here we illustrate the arguments made in the previous section with several numerical examples. In Fig 1 we show two types of adaptive dynamics evolutionary trajectories. When the system is initialized with a single cluster in the first dimension (the cluster was initialized close to the maximum of carrying capacity), it splits into two clusters, which diverge but both remain in the first dimension (left panel). In contrast, when a system has four clusters, which is the maximum steady state level of diversity for a one-dimensional system with the given parameters, a newly formed cluster moves into the second dimension (right panel).

A more complete adaptive dynamics scenario of evolutionary expansion from one to two phenotypic dimensions is shown in the video in S1 Video, in which a system initialized with a single cluster in the first dimension first diversifies to saturation in that dimension, and only then expands into the second dimension. Note that after the first expansion into the second dimension, diversification continues until the available 2-dimensional phenotype space saturates with diversified phenotypic clusters.

To show robustness of these scenarios, we also performed individual-based (S2 Video) and partial differential equation (S3 Video) simulations of the logistic model defined by (6, 7 and 8) (see e.g. [16, 7]). Both types of simulations exhibit the same type of evolutionary dynamics as seen in the adaptive dynamics version: First, the diversity in the existing dimension becomes saturated, thus maximizing the competitive pressure, and only then an expansion into the new dimension occurs. After this initial foray, diversification in two-dimensional space continues until saturation.

### Evolutionary expansion into higher dimensions

**Fig 2:**
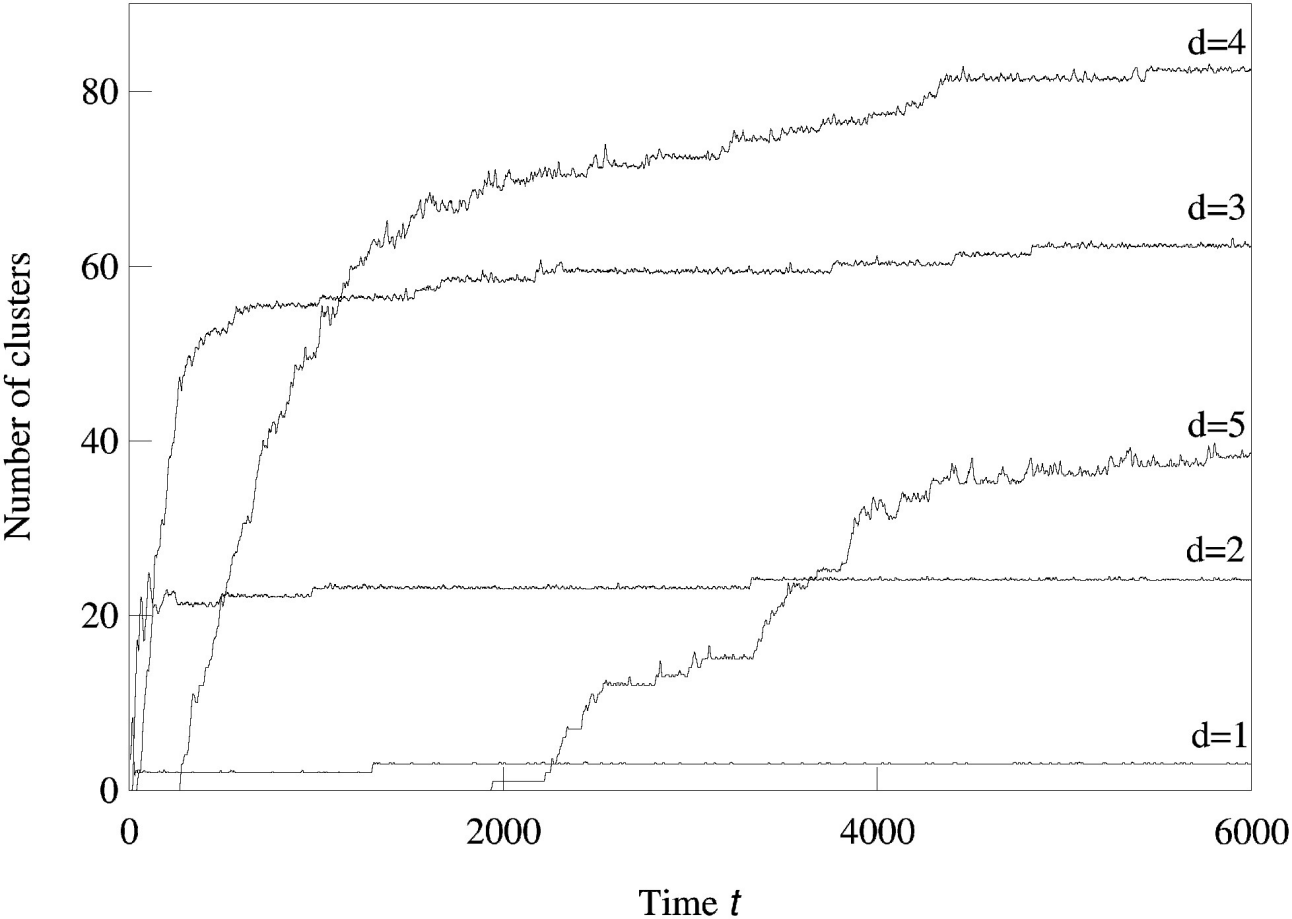
A single run of adaptive dynamics simulation showing sequential competition-driven expansion into higher phenotypic dimensions. The numbers of clusters in 1,2,3,4, and 5-dimensional phenotype spaces are shown as a function of time. Here and in the following, a cluster is considered belonging to the dimension *k* if *x*_*k*_ > 2*σ*_*β*_ and the running average over 10 time units for the number of clusters is shown. The parameters used in the logistic model were *σ*_*β*_ = 0.15, *b* = 0.883, *C* = 1, and *σ*_*α*_ = 0.5.

**Fig 3:**
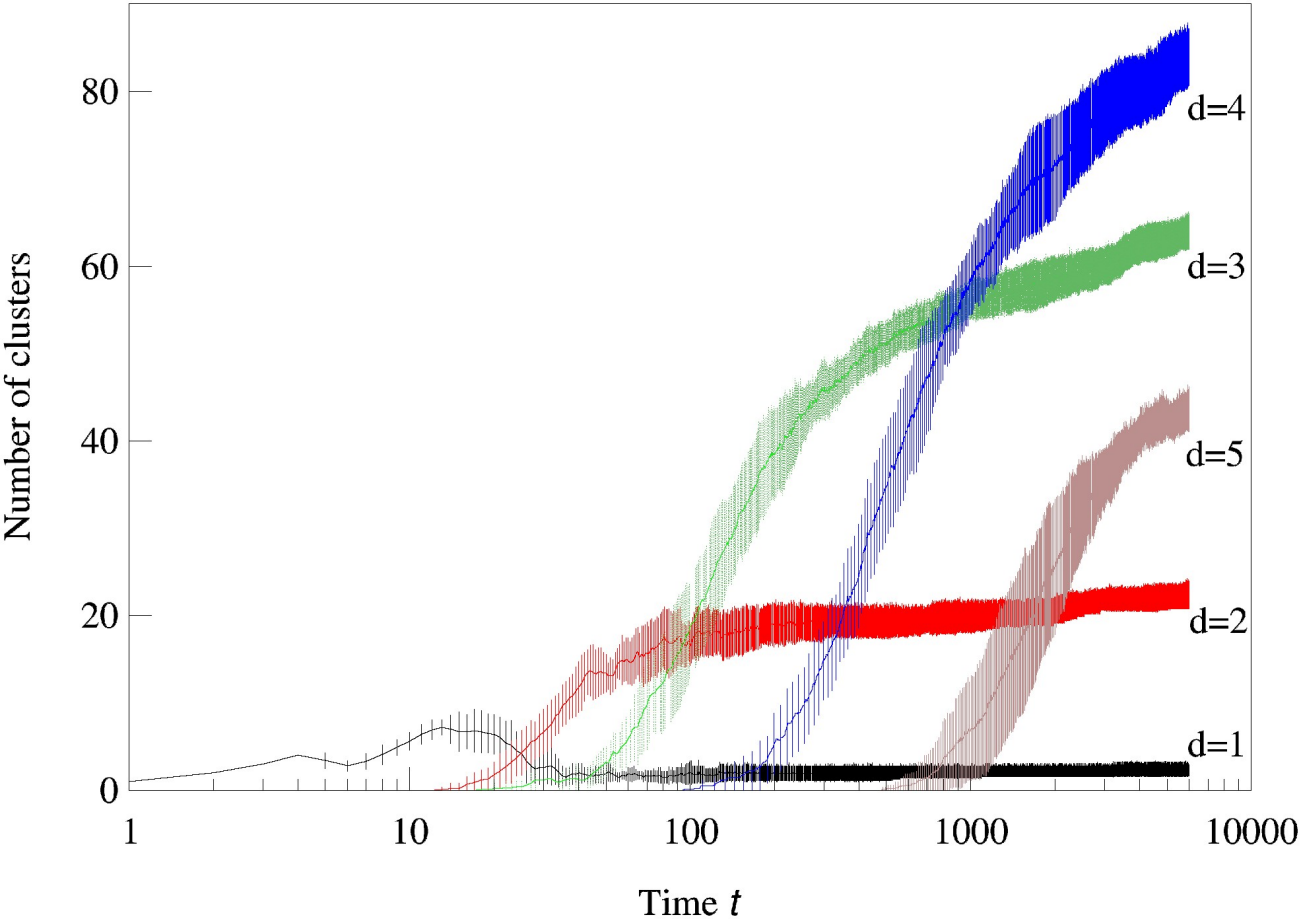
Averaged over 10 runs adaptive dynamics simulations show statistical reproducibility of competition-driven expansion into higher phenotypic dimensions. The numbers of clusters in 1,2,3,4, and 5-dimensional phenotype spaces are shown as a function of time in a semi-log scale. The parameters used in the logistic model were *σ*_*β*_ = 0.17, *b* = 0.9, *C* = 0.9, and *σ*_*α*_ = 0.5.

**Fig 4:**
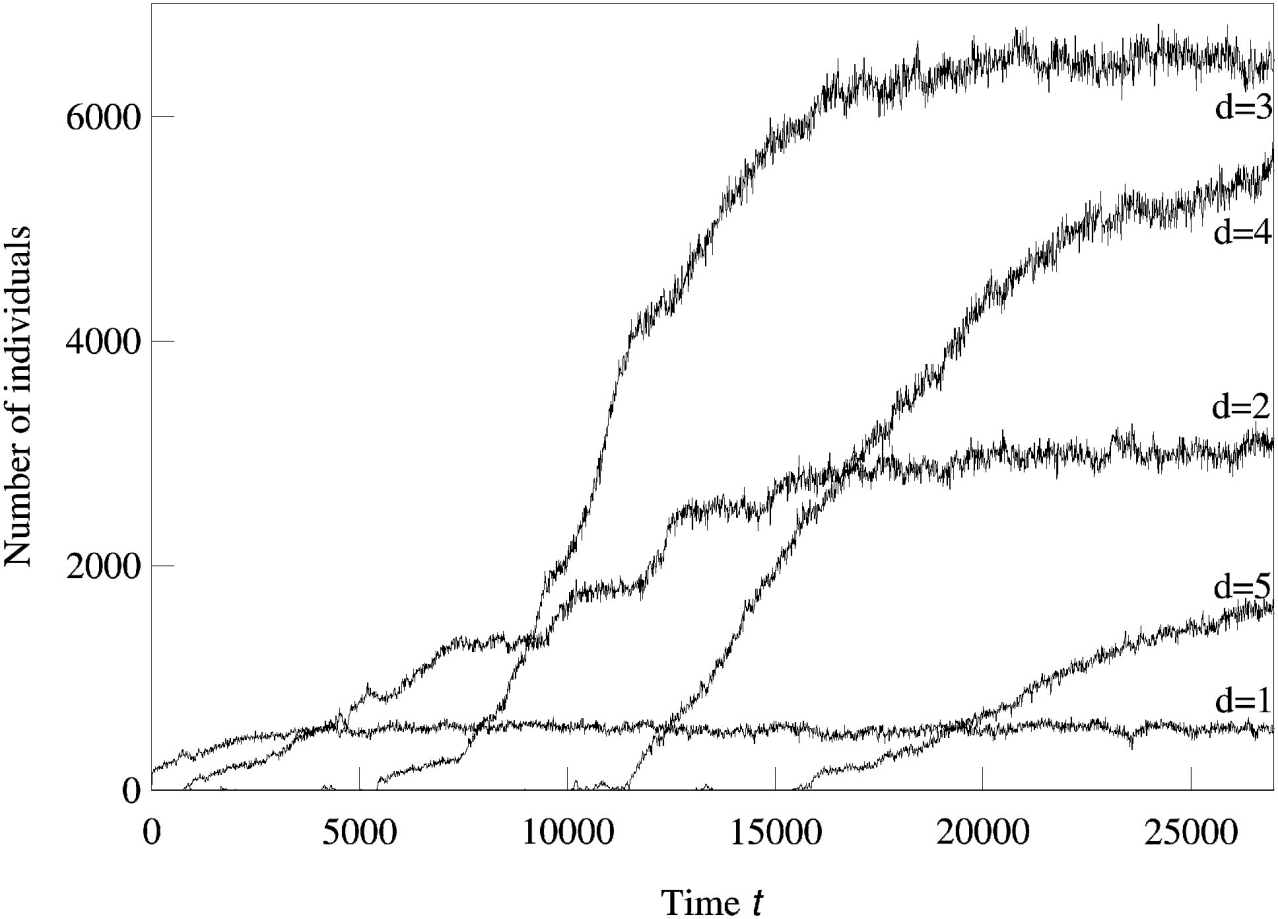
Individual-based simulation showing sequential competition-driven expansion into higher phenotypic dimensions. The numbers of individuals in 1,2,3,4, and 5-dimensional phenotype spaces are shown as a function of time. The parameters used in the logistic model were *σ*_*β*_ = 0.15, *b* = 0.75, *C* = 1, and *σ*_*α*_ = 0.5. The maximum of the carrying capacity *K*_0_ = 500.

We now consider systems in phenotype spaces of dimension larger than two. In our examples we set the highest dimension equal to five. We expect the diversification and acquisition of new dimensions to occur as a sequence of elementary steps similar to the ones presented above for the transition from one to two dimensions. The number of possible diversification options naturally increases due to combinatorics: a population having a one-dimensional phenotype can acquire a two-dimensional phenotype in four possible ways, expanding into dimension 2, 3, 4, or 5. Likewise, there are different ways to expand from 2-dimensional to 3-phenotypes and from 3-dimensional to 4-dimensional phenotypes. The “elementary expansion events” corresponding to these various possibilities do not necessarily happen synchronously due to the stochasticity that is intrinsically present in our adaptive dynamics diversification procedure (and even more so in the individual-based simulations). Hence we do not expect the results to stay precisely as clean as in the case of expansion from 1 into just 2 dimensions. For example, expansion into three dimensional space may happen before all two-dimensional subspaces are completely filled with clusters. Nevertheless, our results shown in Figs 2, 3 and 4 confirm that the general trend remains the same: the expansion into a new dimension, which manifests itself as an appearance of clusters in that dimension, occurs only when a sufficient level of diversification and competition pressure are achieved in the existing dimensions. Fig 3 illustrates the statistical reproducibility of the scenario of expansion into new dimensions, showing results of average over 10 distinct adaptive dynamics runs. In Fig 4 we show the results of individual-based simulations, which exhibit a sequence of expansion events into higher dimensions that is almost identical to that seen in adaptive dynamics.

**Fig 5:**
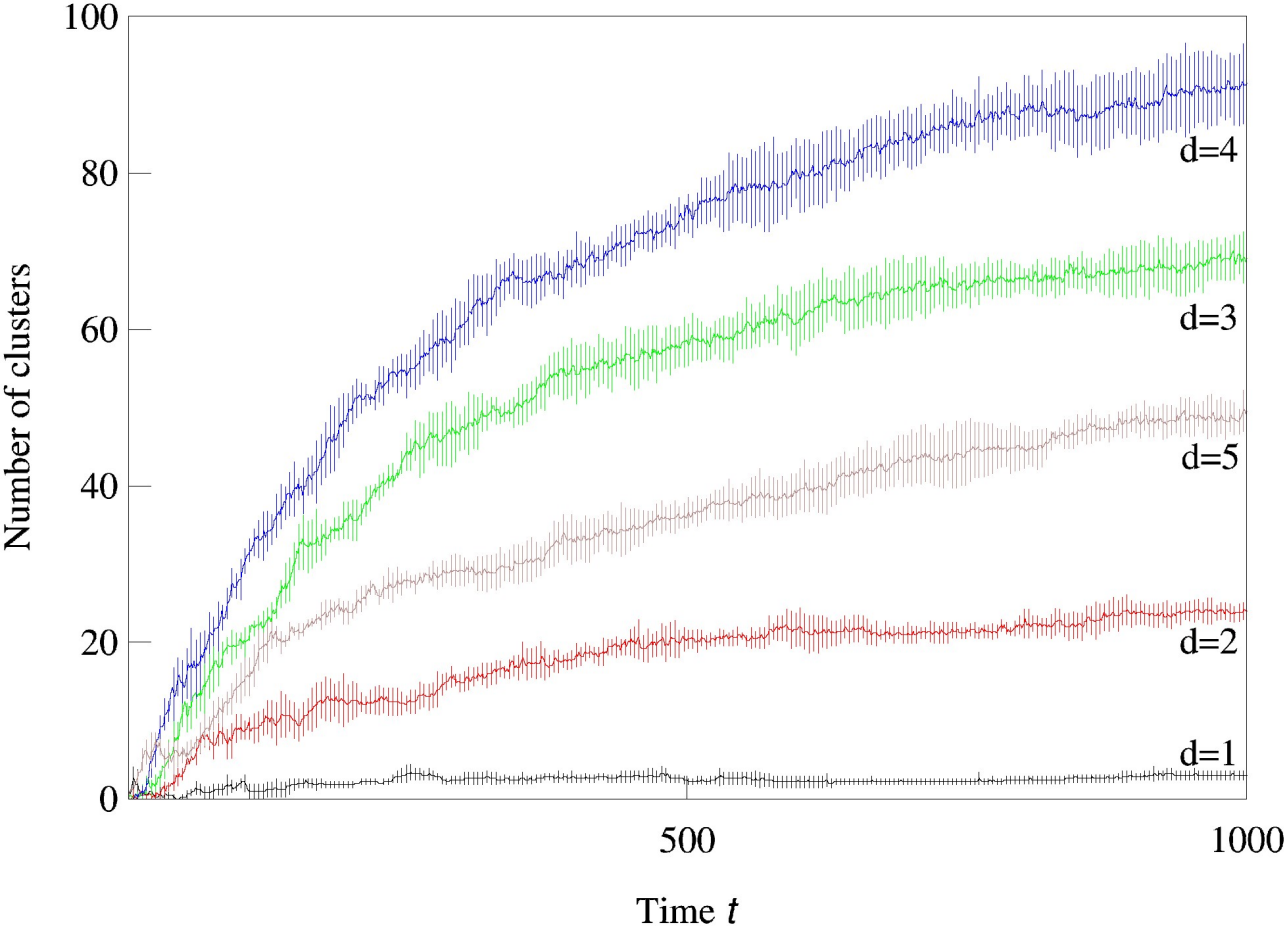
Averaged over 10 runs adaptive dynamics simulation showing that evolutionary expansion occurs simultaneously into all higher dimensions when there is no cost in the birth rate for increased phenotypic complexity. The numbers of clusters in 1,2,3,4, and 5-dimensional phenotype spaces are shown as a function of time. The parameters used in the logistic model were *σ*_*β*_ = 0.15, *b* = 0, *C* = 1, and *σ*_*α*_ = 0.5.

To illustrate that the sequential nature of competition-driven evolutionary expansion into new phenotypic dimensions is indeed conditional on the presence of costs of increased complexity, Fig 5 shows an adaptive dynamics simulation where the cost in the birth rates was set to 0. In this case, expansion occurs simultaneously and instantaneously into all possible phenotypic dimensions, essentially because phenotype expansion only has benefits (both in terms of evading competition and in terms of increasing the carrying capacity). In particular, without costs, the sequential “blunderbuss pattern” [5] is lost.

**Fig 6:**
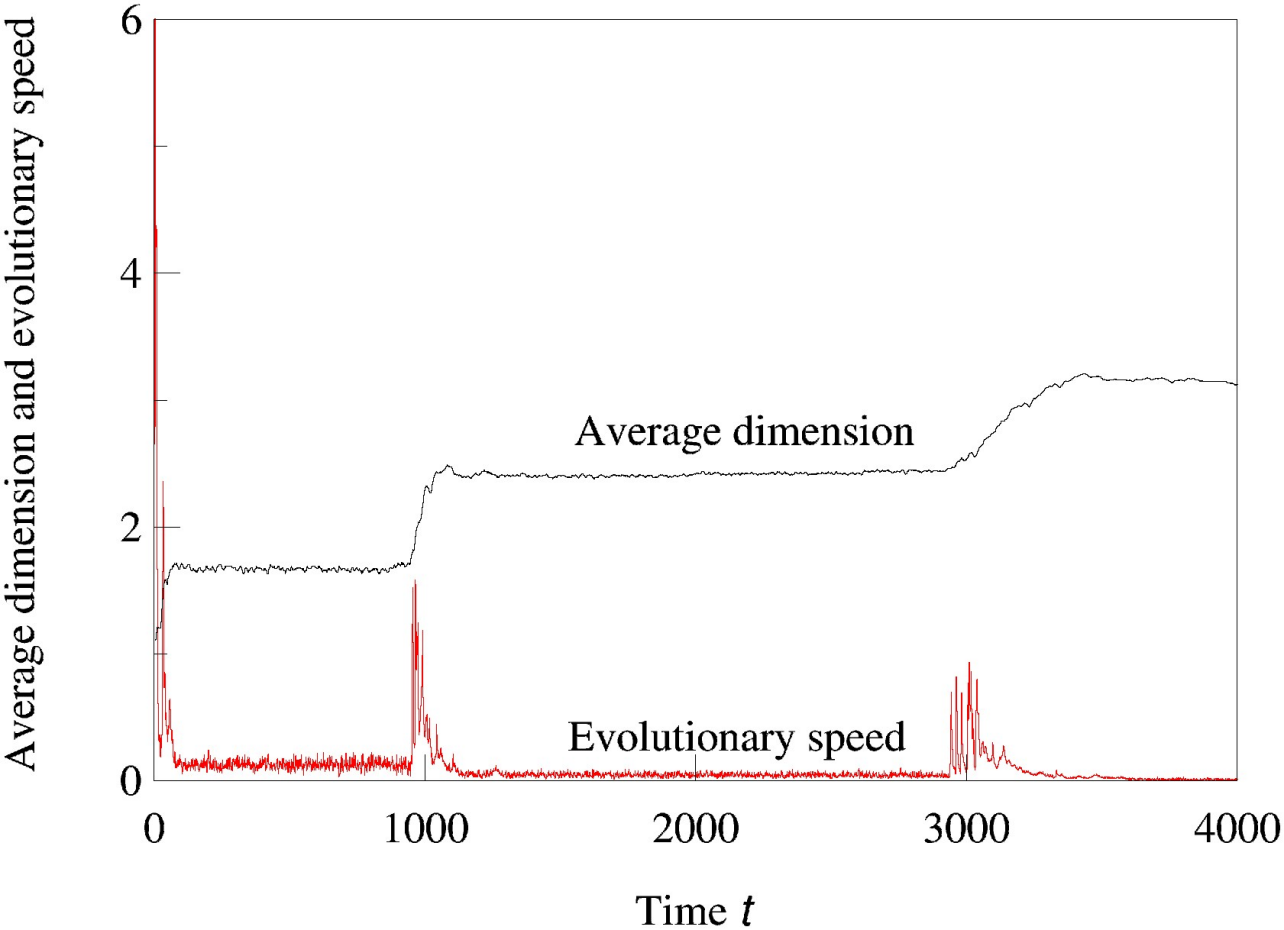
Correlation between bouts of expansion into new phenotypic dimensions and intermittent increases in the average evolutionary speed. The average dimension is computed as the sum of dimensions of all present clusters divided by the number of clusters. The average evolutionary speed is the population-weighted average of the absolute values of the evolutionary speed (measured by the selection gradients given by adaptive dynamics) of all coexisting clusters. (For purposes of illustration, the speed is multiplied by 100.). The parameters used in the logistic model were *σ*_*β*_ = 0.16, *b* = 0.8, *C* = 0.9, *σ*_*α*_ = 0.5, and the maximum dimension was 5.

An interesting consequence of sequential increases in phenotypic complexity as shown in Figs 2 and 3 is that each evolutionary expansion into a new phenotypic dimension opens up a new and initially empty ecological niche. As predicted in [7], these expansions are not only followed by new bouts of diversification, but they also generate an initial increase in the rate of evolution, which subsequently decreases as diversity in the new niche reaches saturation. Thus, sequential increases in complexity lead to intermittent bursts of evolutionary speed, as illustrated in Fig 6.

## Discussion

It seems reasonable and intuitive that evolutionary transitions from simpler to more complex organisms, capable of accessing novel resources or having otherwise novel ecological properties, should go through intermediate phases in which the benefits of novel phenotypes are not fully available, but the cost of developing those phenotypes is already manifest. Cleary, when costs are low, transitions can happen fast, with the rate mostly limited by the speed of accumulation of the necessary mutations. Conversely, when the costs are very high and cannot be compensated by even the fully accessed benefits, the transition may never happen. Here we explored intermediate and arguably more intriguing scenarios, in which costs of increased complexity are high enough to prevent “trivial” transitions due beneficial mutations only, yet once the new phenotype is fully developed, the benefits exceed the costs. We have shown that in such intermediate cases, resource competition can make the difference between success and failure of an evolutionary expansion into a new phenotypic dimension: For low diversity at the existing level of organismic complexity, competitive pressure is weak, and the expansion does not occur, while for high diversity in the existing phenotypic dimensions, competitive pressure becomes strong enough for mutants with higher phenotypic complexity to overcome the costs of increasing complexity.

This intermediate scenario corresponds to a bistability in the invasion fitness landscape with two types of maxima: one type (comprising one or many maxima depending on the amount of diversity) that completely lacks the new phenotypic dimension, while the other type corresponds to the fully developed phenotype in the new dimension. These two types of maxima are separated by a low-fitness area characterized by incomplete benefits but substantial costs. However, it is important to realize that these maxima are not externally imposed, and instead result from the frequency-dependent ecological interactions. When the level of diversification at the existing level of complexity is low, the resulting maximum or maxima are high, and the low-fitness barrier is impassable evolutionarily. But when the level of diversity in the existing dimension becomes saturated, the invasion fitness maxima in that dimension flatten and the barrier to the higher dimension disappears due to competitive pressure from the species living in the lower dimensional phenotype space. Thus, competition enables the crossing into a higher dimensions through an area that is a fitness valley in the absence of competition. It is perhaps helpful to interpret our results in terms of Hutchinsonś niche concept [24]. In that view, niches are dynamically defined by both abiotic factors and competitive pressure from all the coexisting populations. Evolutionary transitions to higher levels of complexity in our models then correspond to an expansion of the niche due to increased diversity, and hence increased competition, in lower-dimensional phenotypes, so that the new niche consists of previously unattained phenotypes in higher dimensions. It may be tempting to interpret our observations in terms of r-K selection paradigm [25], where an acquisition of a new phenotypic dimension makes the species less *r*− and more *K*–strategist. However, in our minds, the evolutionary dynamics displayed in our model are not really related to the problem of *r*− vs *K*–selection. The reason for this is that at any point in time, all coexisting phenotypic clusters, whether complex or simple, are at ecological equilibrium. The dynamics are driven by invasion fitness, but it is not the case that e.g. early on in the evolutionary process r-selected phenotypes are present in the evolving community, whereas later there would be only K-selected phenotypes. Rather, the evolutionary process due to frequency-dependent competition is too complicated to be couched in those terms.

This work is a continuation of our studies of evolutionary dynamics and speciation in multidimensional phenotype spaces. We have previously shown that diversification is more likely with high-dimensional phenotypes [26], that the evolutionary dynamics even of single populations tends to be complicated and possibly chaotic [14], that with complex evolutionary dynamics diversification can occur even if the system does not converge to an evolutionary branching point [16], and that for evolution in given phenotype space, diversification changes a fast evolving community with few species into a saturated multi-species community whose component species are evolving only very slowly [7]. Here we have shown that such an evolutionary standstill may be transient and may be followed by expansion into a new phenotypic dimension, creating a new burst of diversification and subsequent slowdown. Each expansion into a new phenotypic dimension is associated with an increase in the rate of evolutionary changes of phenotypes (evolutionary speed), which overall results in a pattern of intermittent bursts of evolutionary change and diversification on a background of relative stasis (Fig 6).

Putting our results in the context of existing empirical research on the evolutionary increase of organismal complexity appears to be difficult due to a lack of relevant data. Distilling the knowledge about such complexity-expanding transitions from the fossil record is apparently a difficult task, and e.g. determining the timing and the level of organismal complexity associated with fundamental transitions such as the appearance of predation is still debated [27, 28]. Bioinformatics methods have their own difficulties, such as the scarcity of gene annotations, that hinders associating genes with corresponding phenotypic features. Even at a more basic level, separating contributions from biotic and abiotic factors to major evolutionary transitions is a notoriously difficult task (see, for example, the review by [29]), and the role of adaptation in evolutionary increases of complexity is not resolved [9].

Despite all those difficulties, several established evolutionary facts can be viewed as supportive of our conclusion. For example, it has been deduced that the maximum size of organisms has increased mostly in two discrete steps of approximately equal magnitude [30]. Each step required a substantial expansion in organismal complexity: the first step was associated with the appearance of the eukaryotic cell, and the second step with eukaryotic multicellularity. Also, our findings are reminiscent of the notion of rapid adaptive diversification into new adaptive zone as envisioned in [31]. The appearance of new adaptive zones could be linked to an expansion to a higher level of phenotypic complexity, which enables the organisms to function in novel ways and e.g. use novel resources or novel habitats. More generally, many adaptive radiations [6] could be viewed from the perspective of increased organismal complexity allowing for expansion into new regions of phenotype space that subsequently cause bouts of diversification. Adaptive radiations may often be perceived to be driven by geological events, such as the colonization of a new and initially empty habitat (e.g. islands or lakes). But migration leading to such colonizations may itself be driven by ecological pressures in the ancestral habitat. Moreover, there are also cases of adaptive radiation that occur in the absence of geological events, such as the radiation in floral diversity in a group of Solanaceae [32], which apparently occurred in a period without significant geological changes, and instead was likely caused by competition for pollinators. In general, we think that investigating adaptive radiations as ecologically driven increases in phenotypic complexity and subsequent diversification could be a useful perspective.

Finally, a connection could be seen in a number of well-analyzed examples of convergent evolution [33]: multiple appearances of the same potentially complex trait points to selective forces for their origin, and the variation in the timing of the evolution of such traits could indicate that their appearance depends on the presence of the “right” ecological scenario.

Naturally, a lot remains to be explored regarding the fascinating question of the evolution of organismal complexity. We see several immediate possible extensions of our work. First, it would be desirable to have a more realistic representation of competition between individuals that live in distinct sets of dimensions. For example, one should take into account a possible lack of reciprocity in the competitive effects between high-dimensional and low-dimensional individuals. Second, other ecological interactions, such as predation, should be included in future models. In general very little is known about evolution of predator-prey interactions in high-dimensional phenotype spaces. Furthermore, it would be very interesting to investigate evolutionary transitions between different types of ecological interactions (e.g. from competition to cooperation) as a particular form of transition between levels of complexity. Such transitions could also lead to changes in the process of adaptation itself, e.g. due to the appearance of new levels of individuality and multi-level selection. Relaxing the assumption of independent and identical mutational processes in all phenotypic directions could potentially affect our conclusions in several ways. For example, with more complicated mutational processes, previous adaptive changes could by chance create a “platform” that facilitates transition to a higher level of complexity, or emerging constraints could prevent transitions that would otherwise have resulted from ecological pressures. Naturally, our paper does not show in any way that the mechanisms for evolutionary increases in complexity considered here are a prevalent, much less the only mode by which complexity evolves in actual biological systems. Comparing this specific mechanism, which is rather complicated in itself, to other potential mechanisms, such as purely neutral process [34] and assessing likelihoods of different mechanism would appear to be an important future task.

## Supporting information

S1 Video Video of adaptive dynamics of diversification showing that an expansion into the second dimension occurs only once the diversity in the first dimension (horizontal strip at the centre of the frame) becomes saturated with 4 distinct clusters. Parameters are *σ*_*β*_ = 0.15, *b* = 0.85, *C* = 1 and *σ*_*α*_ = 0.5.
(AVI)

S2 Video Video of an individual-based simulation of the logistic equation defined by the birth rate (7), carrying capacity (6) and competition kernel (8). Mutations at birth were implemented as a random offset of the phenotype of the offspring from the ancestral one by ∼ 10^−2^, see [16, 7] for more details. It shows that an expansion into the second dimension occurs only when the diversity in the first dimension (a horizontal strip at the bottom of the frame) reaches 4 distinct clusters. Diversification then continues until the two-dimensional space becomes filled with clusters. The parameters are *σ*_*β*_ = 0.15, *b* = 0.75, *C* = 1 and *σ*_*α*_ = 0.5.
(AVI)

S3 Video Video of a partial differential equation simulation of the logistic equation defined by the birth rate (7), carrying capacity (6) and competition kernel (8). A small diffusion term *D*∇^2^**x** with *D* = 10^−6^ is added to mimic mutations. The simulation illustrates that expansion into the second dimension occurs only when the diversity in the first dimension (a horizontal strip at the bottom of the frame) becomes saturated with an almost continuous phenotype distribution concentrated around three clusters. The parameters are *σ*_*β*_ = 0.15, *b* = 0.35, *C* = 1 and *σ*_*α*_ = 0.5.
(AVI)
